# Supplementary material for: Molecular Characterization of WCK 5222 (Cefepime/Zidebactam)-Resistant Mutants Developed from a Carbapenem-Resistant Pseudomonas aeruginosa Clinical Isolate
Source: Microbiol Spectr. 2022 Feb 23;10(1):e02678-21. doi: 10.1128/spectrum.02678-21 (PMC8865557; doi:10.1128/spectrum.02678-21)
Supplement: SUPPLEMENTAL FILE 1 — Supplemental material. Download SPECTRUM02678-21_Supp_1_seq6.pdf, PDF file, 1.3 MB [file spectrum02678-21_supp_1_seq6.pdf]

Fig. S1

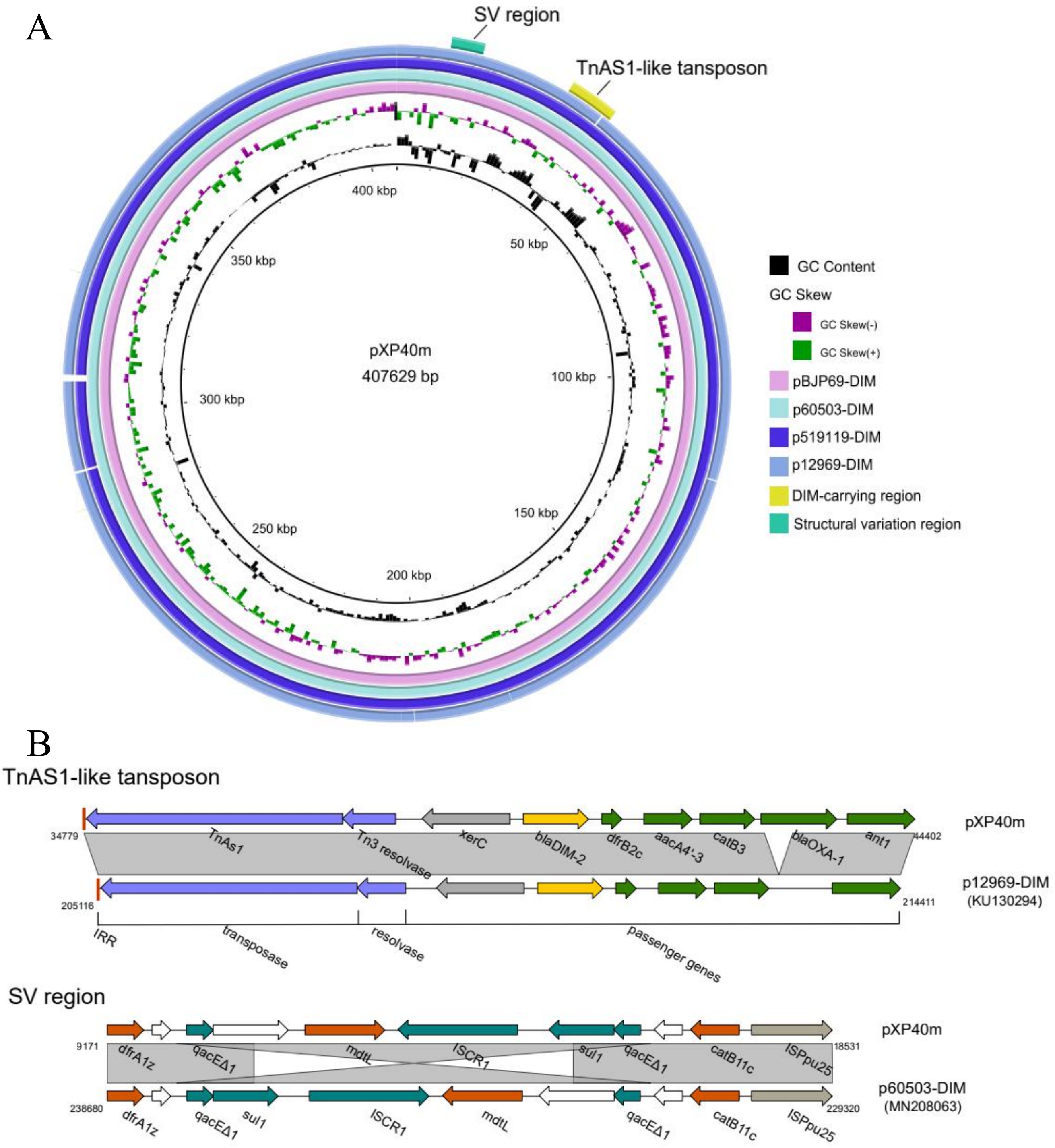

Fig. S1 Schematic maps of the plasmid pXP40m and its accessory modules. (A) Genetic organization of pXP40m and comparison with similar plasmid backbones. Structure variation region (green) and the *bla*$_{\text{DIM-1}}$-carrying region (yellow) are represented in the outermost circles. Homology regions are represented in blue grey, blue, light blue and pink for the plasmids p12969-DIM, p519119-DIM, p60503-DIM and pBJP69-DIM, respectively. The inner multi-peak circle illustrates GC skew +/- and GC content. The innermost circle indicates the scale. (B) Upper: The *bla*$_{\text{DIM-2}}$-carrying genetic element. Yellow and green arrows represent the *bla*$_{\text{DIM-2}}$ gene and other resistant genes, respectively. Blue arrows represent the transposase (*TnAs1*) and resolvase (*Tn3*) genes. Grey arrows represent the *xerC* gene. IRR, inverted repeat right. Lower: The structure variation region of plasmid pXP40m compared with plasmid p60503-DIM.

Fig. S2

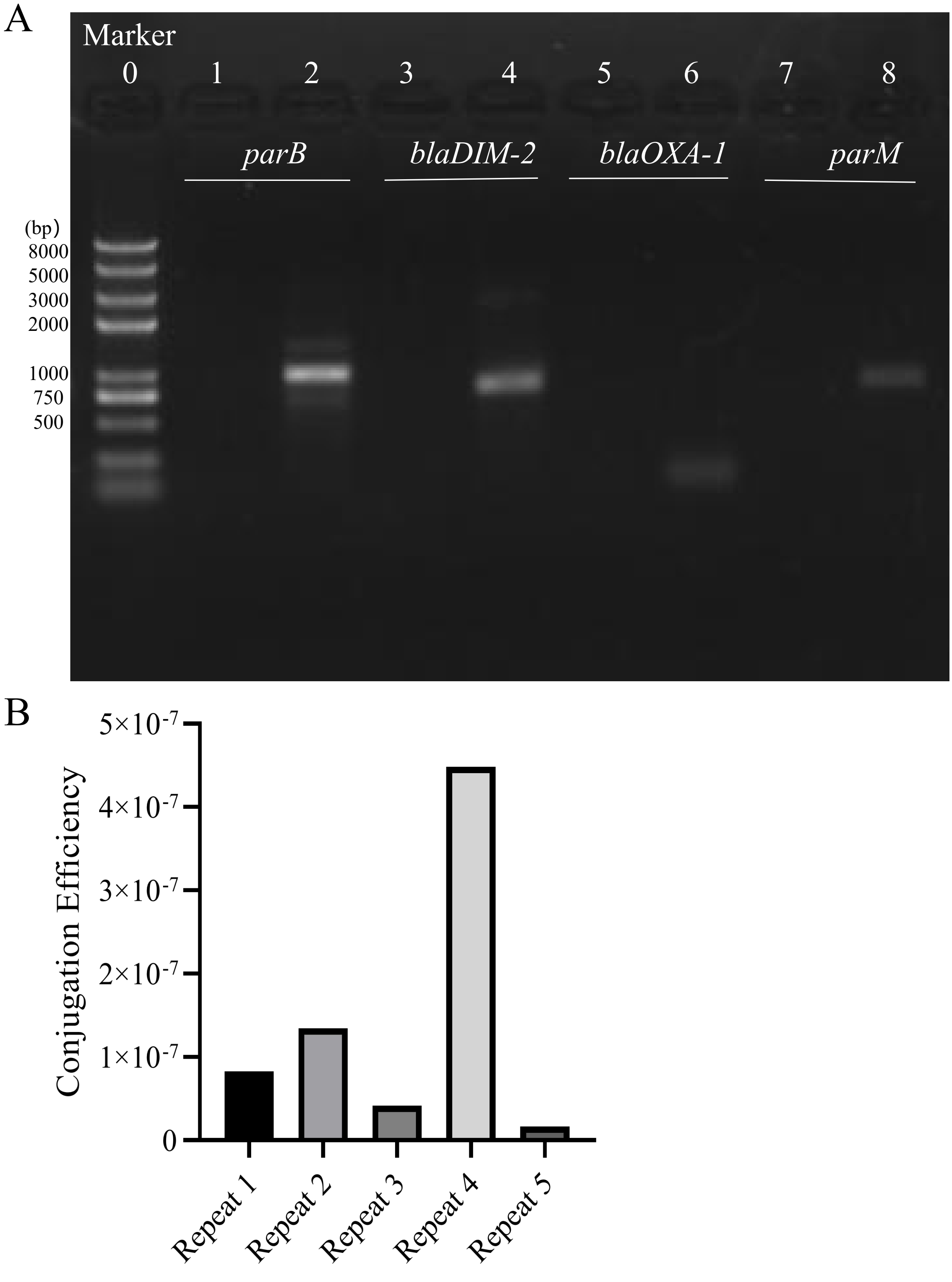

Fig. S2 Transconjugant verification and conjugation frequency. (A) Verification of the presence of genes carried by pXP40m. The presence of *parB* (Lane 1, 2), $bla_{\text{DIM-2}}$ (Lane 3, 4), $bla_{\text{OXA-1}}$ (Lane 5, 6) and *parM,* (Lane 7, 8) that are localized on pXP40m in the indicated strains were determined by PCR with primers that listed in Table S1. PAO1 and the transconjugant were subjected to plasmid isolation and the extracted DNA was used as the templates for PCR. Lanes 1, 3, 5, 7, DNA isolated from PAO1; Lanes 2, 4, 6, 8, DNA isolated from the transconjugant. (B) Five independent conjugation experiments were performed to determine the conjugation frequency by using NKPa-71 strain as the donor strain and PAO1 carrying P_{prtR}-*lacZ* as the recipient strain.

Fig. S3

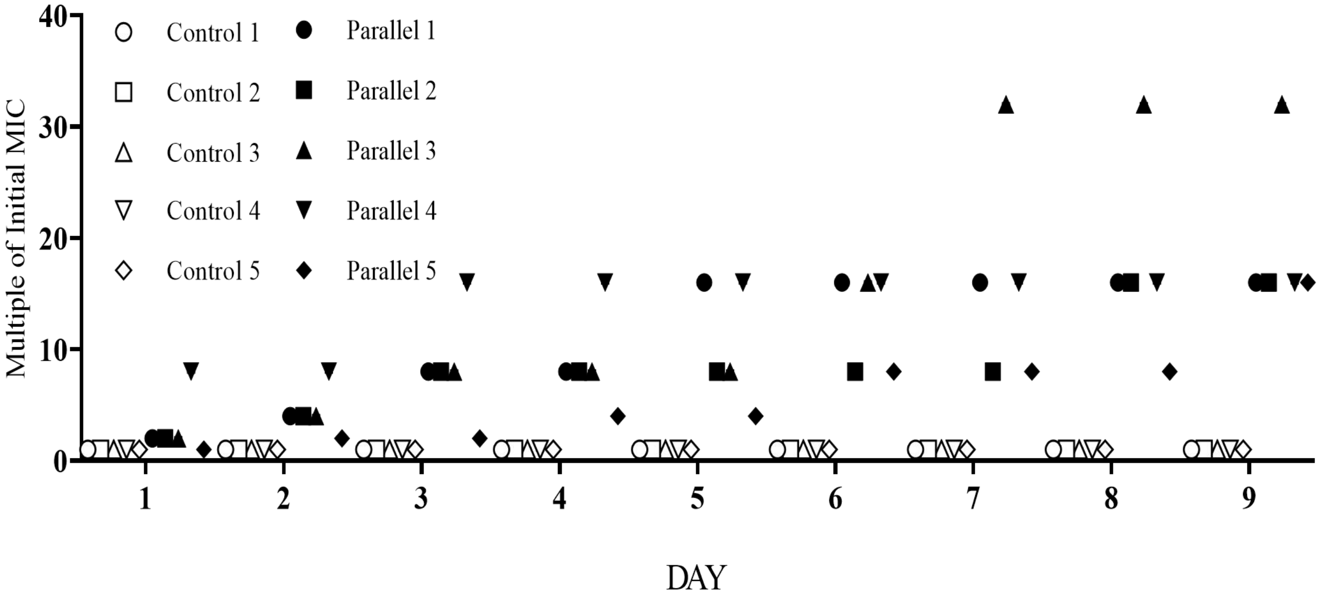

Fig. S3 Resistance development of NKPa-71 to WCK 5222. MICs of the passaged populations were determined.

Fig. S4

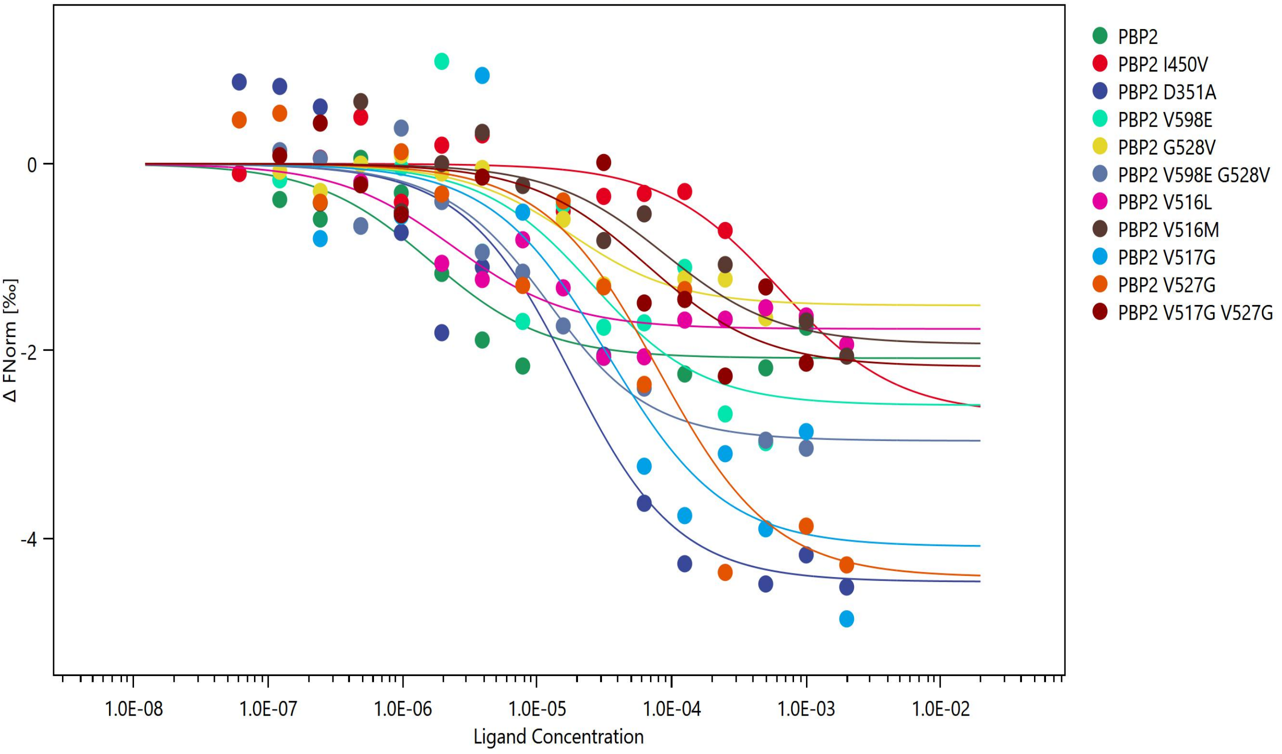

Fig. S4 Affinities of the PBP2 protein mutations and zidebactam. MST analyses of the interactions between the mutated PBP2 proteins and zidebactam. The dissociation constant of each pair was listed in Table 5.

Fig. S5

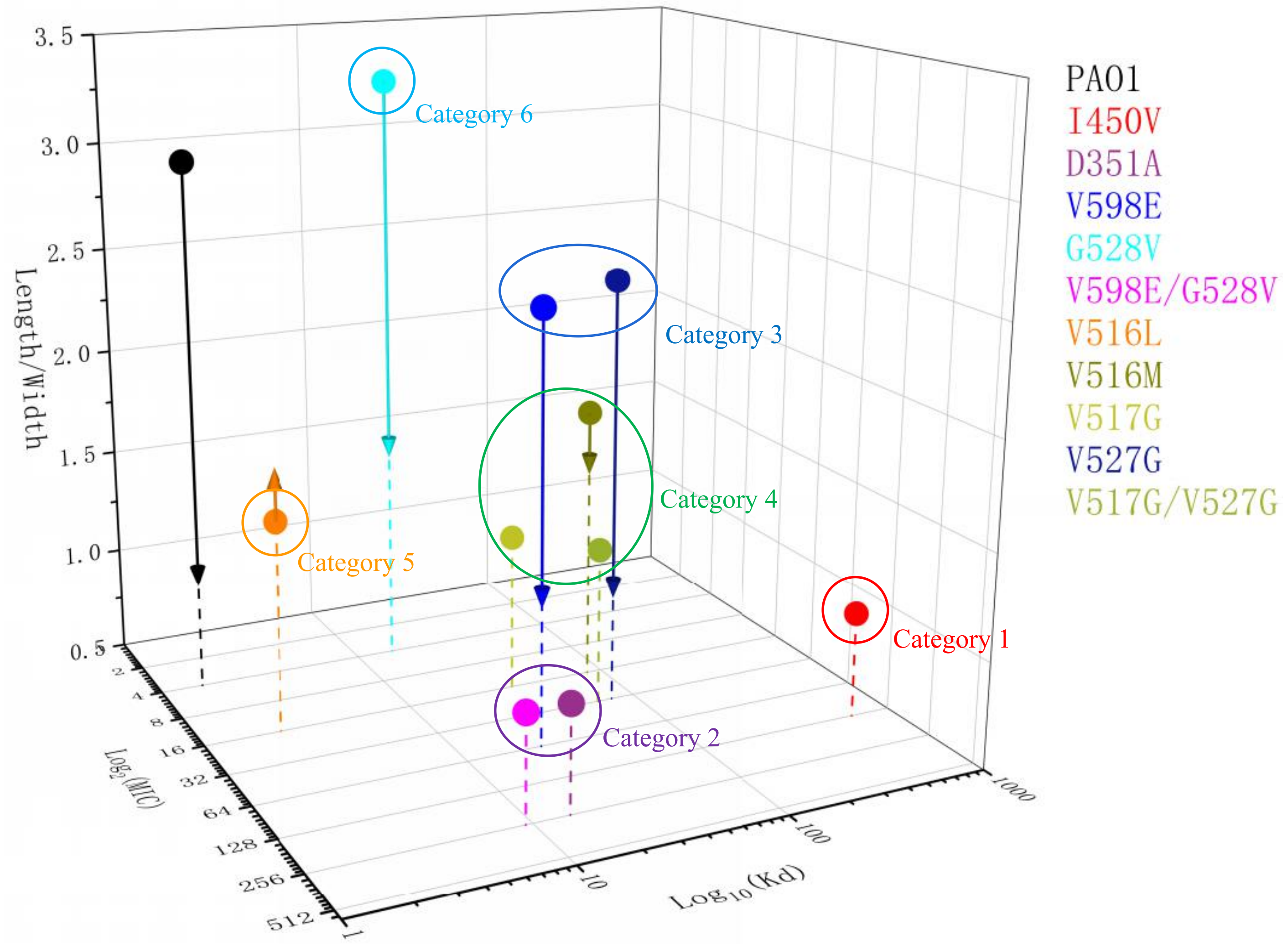

Fig. S5 Characterizations of the PBP2 mutations. A three-dimensional graph shows categories of the PBP2 mutations based on the influences on resistance levels (MICs), bacterial morphology (length/width) and affinity to zidebactam (Kd). The arrows indicate the morphological changes before and after zidebactam treatment. The dashed lines indicate the exact positions on the bottom.

Fig. S6

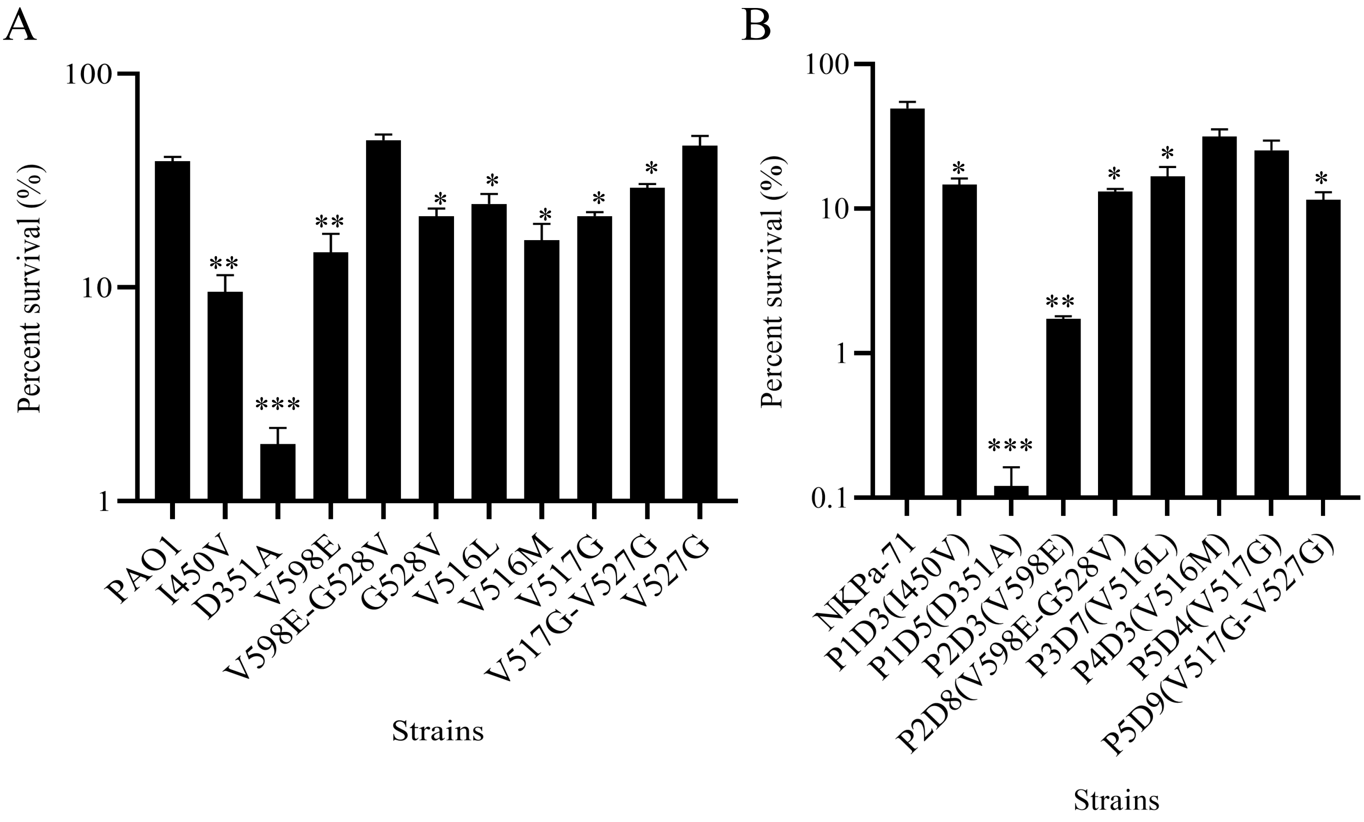

Fig. S6 Bacterial survival rates following hypoosmatic shock. PAO1 and mutants (A) or NKPa-71 and evolutional isolates (B) at the OD_{600} of 1.0 were collected and resuspended in 150 mM NaCl or ddH_2O for 15 min. The live bacteria numbers were determined by serial dilution with LB and plating. The survival rate was calculated by dividing the live bacteria number in ddH_2O by the corresponding live bacteria number in the NaCl solution. *, P<0.05; **, P<0.01; ***, P<0.001 compared to the wild type PAO1 by Student's t-test.

Table S1 Resistance genes carried by NKPa-71.

Resistance gene	Location	Function
catB7	genome	Phenicol resistance
aph(3')-IIb	genome	Aminoglycoside resistance
blaPAO	genome	β-lactam resistance
blaOXA-395	genome	β-lactam resistance
fosA	genome	Fosfomycin resistance
crpP	genome	β-lactam resistance
qnrVC6	plasmid	Fluoroquinolone resistance
aac(6')-Ib-cr	plasmid	Fluoroquinolone and aminoglycoside resistance
sul1	plasmid	Sulphonamide resistance
catB3	plasmid	Phenicol resistance
aph(6)-Id	plasmid	Aminoglycoside resistance
aadA1	plasmid	Aminoglycoside resistance
aph(3'')-Ib	plasmid	Aminoglycoside resistance
aac(6')-IIa	plasmid	Aminoglycoside resistance
aac(6')-Ib-Hangzhou	plasmid	Aminoglycoside resistance
blaOXA-4	plasmid	β-lactam resistance
blaDIM-2	plasmid	β-lactam resistance
dfrA1	plasmid	Trimethoprim resistance
dfrB7	plasmid	Trimethoprim resistance

Table S3. Mutation sites of PBP2 in the evolved strains.

Strains	Parallels	Days	Mutation sites
P1D3	1	3	I450V
P1D5	1	5	D351A
P2D3	2	3	V598E
P2D8	2	8	V598E, G528V
P3D3	3	3	No Mutation
P3D6	3	6	V516L
P3D7	3	7	V516L

P4D1	4	1	V516M
P4D3	4	3	V516M
P5D2	5	2	No Mutation
P5D4	5	4	V517G
P5D6	5	6	V517G, V527G
P5D9	5	9	V517G, V527G

Table S4. Bacterial strains, plasmids and primers used in this study.

Strain/ Plasmid /Primer	Description	Source (Reference)
P. aeruginosa		
NKPa-71	Clinical isolate *Pseudomonas aeruginosa*	This study
PAO1	Wild type strain of *Pseudomonas aeruginosa*	(1)
PA14	Wild type strain of *Pseudomonas aeruginosa*	(1)
PAO1/pXP40m	PAO1 conjugated with NKPa-71	This study
PA14/pXP40m	PA14 conjugated with NKPa-71	This study
PAO1 /Tn7T-*PbpA*	PAO1 with *PbpA* inserted on chromosome with mini-Tn7T insertion; Gm^r	This study
△*PbpA*/Tn7T-*PbpA*	△*PbpA* with *PbpA* inserted on chromosome with mini-Tn7T insertion; Gm^r	This study
PAO1/pUCP24--*blaDIM-2*	PAO1 with *blaDIM-2* cloned from pXP40m	This study
PA14/pUCP24--*blaDIM-2*	PA14 with *blaDIM-2* cloned from pXP40m	This study
P. putida (K2440)	Wild type strain of *Pseudomonas putida*	This study
P. putida /pXP40m	*P. putida* conjugated with NKPa-71	This study
**Plasmid**		
pEX18Tc	Gene replacement vector; Tc^r, *oriT*$^+$, *sacB*$^+$	(2)
pUC18T-mini-Tn7T-Gm	mini-Tn7 base vector from insertion into chromosome attTn7 site; Gm^r	(2)
pUCP24	Gene cloning, Gm^r	This study
pET28a	Protein expression	This study
pUC18T-mini-Tn7T-Gm-*PbpA*	pUC18T-mini-Tn7T-Tc with *PbpA*; Gm^r	This study
pEX18Tc-△*PbpA*	*PbpA* gene of PAO1 deletion on pEX18Tc; Tc^r	This study
pUCP24--*blaDIM-2*	*blaDIM-2* gene cloning	This study
pET28a-PBP2	PBP2 protein expression	This study
pXP40m	Megaplasmid in NKPa-71	This study
**Primer**	**Sequence (5'→3')**	**Function**
DIM-2 F	CGAGCTCTTAGAGGGAAAAATCGAATGAGA	*blaDIM-2* cloning and verification
DIM-2 R	CGGATCCGCCTCAATCAGCCGACGC	*blaDIM-2* cloning and verification

OXA-1 F	GCCCTTTACCAAACCAATACTT	*blaOXA-1* verification
OXA-2 R	TCGCATTTTTCTTGGCTTTTA	*blaOXA-1* verification
ParB F	TTGAAGAAAGGCGGTCGTC	*parB* verification
ParB R	AGCCATCGGTCTCCATCG	*parB* verification
PbpA UPF	CGGAATTCGCTCCGTGCGTTTCC	deletion of *pbpA*
PbpA UPR	CTGTTCAAGGGCGGGTCGGGACTGGGG ACTATTTG	deletion of *pbpA*
PbpA DNF	AGTCCCCAGTCCCGACCCGCCCTTGAAC AGTAAC	deletion of *pbpA*
PbpA DNR	CCAAGCTTGCCAGCAGTTTGCCGTAGA	deletion of *pbpA*
PbpA F	CGAGCTCACAAATAGTCCCCAGTCCCG	*pbpA* cloning
PbpA F	CAAGCTTGAAGTTACTGTTCAAGGGCGG	*pbpA* cloning
EX -40PBP2F	CATGCCATGGACCACCTGCAGG	PBP2 expression without Signal peptide
EX -40PBP2R	CCAAGCTTCTGTTCAAGGGCGGGCG	PBP2 expression without Signal peptide

References

1. Liberati, N.T., Urbach, J.M., Miyata, S., Lee, D.G., Drenkard, E., Wu, G., Villanueva, J., Wei, T. and Ausubel, F.M. (2006) An ordered, nonredundant library of Pseudomonas aeruginosa strain PA14 transposon insertion mutants. *Proc Natl Acad Sci U S A*, **103**, 2833-2838.
2. Choi, K.-H. and Schweizer, H.P. (2006) mini-Tn7 insertion in bacteria with single attTn7 sites: example Pseudomonas aeruginosa. *Nat Protoc*, **1**, 153-161.
